# On-ward participation of a hospital pharmacist in a Dutch intensive care unit reduces prescribing errors and related patient harm: an intervention study

**DOI:** 10.1186/cc9278

**Published:** 2010-10-04

**Authors:** Joanna E Klopotowska, Rob Kuiper, Hendrikus J van Kan, Anne-Cornelie de Pont, Marcel G Dijkgraaf, Loraine Lie-A-Huen, Margreeth B Vroom, Susanne M Smorenburg

**Affiliations:** 1 Department of Hospital Pharmacy, Academic Medical Center, Meibergdreef 9, 1105 AZ Amsterdam, The Netherlands; 2 Department of Intensive Care, Academic Medical Center, Meibergdreef 9, 1105 AZ Amsterdam, The Netherlands; 3 Department of Clinical Epidemiology and Biostatistics, Academic Medical Center, Meibergdreef 9, 1105 AZ Amsterdam, The Netherlands; 4 Department of Quality and Process Innovation, Academic Medical Center, Meibergdreef 9, 1105 AZ Amsterdam, The Netherlands

## Abstract

**Introduction:**

Patients admitted to an intensive care unit (ICU) are at high risk for prescribing errors and related adverse drug events (ADEs). An effective intervention to decrease this risk, based on studies conducted mainly in North America, is on-ward participation of a clinical pharmacist in an ICU team. As the Dutch Healthcare System is organized differently and the on-ward role of hospital pharmacists in Dutch ICU teams is not well established, we conducted an intervention study to investigate whether participation of a hospital pharmacist can also be an effective approach in reducing prescribing errors and related patient harm (preventable ADEs) in this specific setting.

**Methods:**

A prospective study compared a baseline period with an intervention period. During the intervention period, an ICU hospital pharmacist reviewed medication orders for patients admitted to the ICU, noted issues related to prescribing, formulated recommendations and discussed those during patient review meetings with the attending ICU physicians. Prescribing issues were scored as prescribing errors when consensus was reached between the ICU hospital pharmacist and ICU physicians.

**Results:**

During the 8.5-month study period, medication orders for 1,173 patients were reviewed. The ICU hospital pharmacist made a total of 659 recommendations. During the intervention period, the rate of consensus between the ICU hospital pharmacist and ICU physicians was 74%. The incidence of prescribing errors during the intervention period was significantly lower than during the baseline period: 62.5 per 1,000 monitored patient-days versus 190.5 per 1,000 monitored patient-days, respectively (*P *< 0.001). Preventable ADEs (patient harm, National Coordinating Council for Medication Error Reporting and Prevention severity categories E and F) were reduced from 4.0 per 1,000 monitored patient-days during the baseline period to 1.0 per 1,000 monitored patient-days during the intervention period (*P *= 0.25). Per monitored patient-day, the intervention itself cost €3, but might have saved €26 to €40 by preventing ADEs.

**Conclusions:**

On-ward participation of a hospital pharmacist in a Dutch ICU was associated with significant reductions in prescribing errors and related patient harm (preventable ADEs) at acceptable costs per monitored patient-day.

**Trial registration number:**

ISRCTN92487665

## Introduction

Since the publication of the report *To Err is Human *[1], medical errors have been of major concern worldwide. A systematic review of medical record studies on adverse events showed that the median overall incidence of in-hospital adverse events was 9.2%, with a median percentage of preventability of 43.5%. Surgical-related events (39.6%) and medication-related events (15.1%) constituted the majority of adverse events [2]. A retrospective record review study in 21 hospitals in The Netherlands demonstrated that the national incidence of adverse events - after weighting for the sampling frame - was 5.7%, of which 2.3% were preventable. More than 15% of all adverse events were related to medication, of which 21.2% were considered preventable [3].

Patients admitted to an intensive care unit (ICU) are at high risk for medication errors and related patient harm (preventable adverse drug events (preventable ADEs)), due to the critical nature of their illnesses, polypharmacy, use of high-risk drugs, and a high frequency of changes in pharmacotherapy [4-10]. Several studies have shown that on-ward, daily participation of a clinical pharmacist in the ICU can effectively and efficiently reduce the number of medication errors and related patient harm [11-23]. The number of medication errors was reduced threefold to fivefold but this required half-time, or even full-time (40 hours per week), commitment of a clinical pharmacist to the ICU patient care team [11,12].

In The Netherlands, the staff of a hospital pharmacy consists in general of hospital pharmacists and residents; there are currently no posts for clinical pharmacists specialized in on-ward activities. Dutch hospital pharmacists are scarce (on average, 0.75 hospital pharmacists are available per 100 hospital beds, compared with 1.42 in the United Kingdom and 14.1 in the USA [24,25]) and back-office activities (such as quality assurance of sterile product compounding, therapeutic drug monitoring, medication logistics) take up most of the hospital pharmacist's time. This type of hospital pharmacy organization model limits the clinical activities to centralized off-ward services such as control of drug dosages and interactions and an on-call duty for consultations (a passive approach).

For these reasons, we cannot directly transfer the successful intervention programs of Leape and colleagues [11] or Kaushal and colleagues [12] to the Dutch hospital setting. Such programs would require a comprehensive and daily on-ward participation of a hospital pharmacist in an ICU. Within the current organization model of the hospital pharmacy in The Netherlands, such participation is not feasible because it is too time-consuming. Given the increasing awareness of medication safety problems in The Netherlands [3,26,27], however, a proactive on-ward involvement of Dutch hospital pharmacists (an active approach) seems desirable.

We therefore designed an on-ward participation program for a hospital pharmacist that was tailored to our specific setting, and conducted an intervention study to explore whether this program could be of added value to medication safety in a Dutch ICU. Our main research questions were: is the designed program associated with a reduction in prescribing errors and related patient harm?, can the study results increase the efficiency of the designed program in the future?, and what are the additional costs of the designed program considering the intensified contribution of a hospital pharmacist in an ICU?

## Materials and methods

### Design and setting

The study was performed in the adult medical and surgical ICU of the Academic Medical Centre, a 1,002-bed (tertiary-care) academic hospital in Amsterdam. The medical staff of the closed-format, 28-bed ICU consisted of board-certified intensivists, ICU fellows and residents. Residents, mainly from the Department of Anesthesiology and the Department of Internal Medicine, received 6 months of training in the ICU department and rotated out every 6 months (October and April).

The study was divided into two periods: a baseline period (3 weeks) and an intervention period (8 months). In addition, the intervention period was subdivided into two halves to determine whether outcome measures were influenced by a learning process over time.

Before the start of the study and during the baseline and intervention periods, the clinical services, including the ICU, offered by our central hospital pharmacy department were on-call availability of a hospital pharmacist or hospital pharmacy resident for consultations and therapeutic drug monitoring. Furthermore, a decentralized pharmacy satellite located in and dedicated solely to the care of patients on the ICU offered services consisting of preparation of ready-to-use parenteral medication by pharmacy technicians. The prepared parenteral medication orders were verified twice a day in the central hospital pharmacy department by a hospital pharmacist. All other medication orders were not routinely verified. The ICU was equipped with an electronic ICU patient data management system (PDMS) (Metavision^®^; iMDSoft, Sassenheim, The Netherlands). This PDMS offers a minute-by-minute collection and displays various vital patient parameters, laboratory values and data from medical devices, and also presents patient information such as treatment policy and drug regimen. The incorporated electronic prescribing module was not equipped with a clinical decision support system. The PDMS was also not accessible from the central hospital pharmacy department.

Two hospital pharmacists (RK and HJvK), with more than 10 years of hospital practice experience, were assigned to the designed program to guarantee continuity and quality of the intervention (further referred to as ICU hospital pharmacists). These two ICU hospital pharmacists did not rotate in the clinical services schedule offered by the central hospital pharmacy department. Before the start of the study, both ICU hospital pharmacists completed a training period of 4 weeks in the ICU. During this training, they familiarized themselves with the daily practices and routines in the ICU ward and the prevailing medication protocols and guidelines, and they learned how to retrieve all relevant information from PDMS.

### Study population

All patients admitted to the ICU between 3 October 2005 and 30 June 2006 were included in the study. If a patient was both admitted and subsequently discharged on days when the ICU hospital pharmacist was absent from the ward, the related patient-days and medication orders were not taken into account for the result calculations. No exclusion criteria were applied.

The research protocol was submitted for consideration to the Medical Ethics Committee of the Academic Medical Center before the start of the study. This Medical Ethics Committee judged the protocol as not needing approval. The present research investigates the influence of an intervention aimed at quality improvement of the medication-prescribing process. The integrity of the patient is therefore not influenced by the intervention and, according to the Dutch Medical Ethics Law, the study is not subjected to medical ethical approval. All data were collected anonymously.

### Activities during the baseline period and data collection

During the baseline period, the ICU hospital pharmacists collected data on the ICU. The data were collected after the daily patient care round but prior to the daily multidisciplinary patient review meeting on the ICU. Only one senior ICU staff member (A-CdP) was informed about the presence of the ICU hospital pharmacists on the ICU ward. A private room with a PDMS computer was made available. The ICU hospital pharmacist evaluated each new medication order for its appropriateness for given indication, duration of therapy, drug dosage and frequency, risk of drug-drug and drug-disease interactions; the medication scheme as whole was checked for pharmacological duplications and drug omissions. Medications prescribed on days when the ICU hospital pharmacist was absent from the ICU ward were reviewed retrospectively on the subsequent monitoring day. The international and national pharmacotherapy guidelines and local evidence-based pharmacotherapy protocols were used for this evaluation.

For each detected prescribing issue, the ICU hospital pharmacist recorded the date, patient characteristics (age, sex, weight, Acute Physiology and Chronic Health Evaluation (APACHE) II score calculated by the PDMS, and admission type (acute or elective)), medication details and the pharmacist's recommendation. For ethical reasons, these recommendations were discussed with A-CdP. If consensus was reached between the ICU hospital pharmacists and A-CdP, the medication orders were corrected by A-CdP and the ICU hospital pharmacist scored the related prescribing issue as a prescribing error.

**Figure 1:**
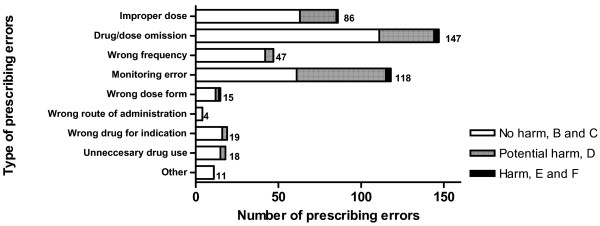
**Type, incidence and severity of prescribing errors found by intensive care unit hospital pharmacists during the whole study period**. Data for prescribing errors found by intensive care unit hospital pharmacists during the whole study period. The severity was scored according to The National Coordinating Council for Medication Error Reporting and Prevention Taxonomy of Medication Errors (categories B to F). The monitoring error category consists of the following types of prescribing errors: wrong dose according to therapeutic drug monitoring, wrong dose according to laboratory tests, organ function or renal replacement therapy requirements, drug-disease interaction, drug-drug interaction, pharmacologic duplications, unrecognized adverse drug reactions.

Subsequently, prescribing errors were categorized by type (Figure 1) and by severity at the time of detection (Table 1), according to The National Coordinating Council for Medication Error Reporting and Prevention (NCC-MERP) classification [28]. If patient harm occurred, the Common Terminology Criteria for Adverse Events criteria (version 3.0) were used to objectively grade the magnitude of harm. According to these criteria, patient harm was categorized as mild, moderate, severe, life-threatening or leading to death [29].

**Table 1:** Severity of medication errors

Major divisions	Subcategory	Description
Error, no harm	Category B	Error did not reach the patient, because it was intercepted before or during administration process
	Category C	Error reached the patient but did not cause patient harm
Error, potential preventable ADE	Category D	Error reached the patient and required monitoring to confirm that it resulted in no harm to the patient and/or required intervention to preclude harm
Error, preventable ADE	Category E	Error may have contributed to or resulted in temporary harm to the patient and required intervention
	Category F	Error may have contributed to or resulted in temporary harm to the patient and required initial or prolonged hospitalization
	Category G	Error may have contributed to or resulted in permanent patient harm
	Category H	Error required intervention necessary to sustain life
	Category I	Error may have contributed to or resulted in the patient's death

Adapted from The National Coordinating Council for Medication Error Reporting and Prevention Taxonomy of Medication Errors [28]. ADE, adverse drug events.

The initial classification of the prescribing error type (grouping into a NCC-MERP category) was performed by the ICU hospital pharmacist who detected the prescribing error. The final classification was performed together with the other ICU hospital pharmacist to assure validity of the interpretation.

### Activities during the intervention period and data collection

During the intervention period, all attending ICU physicians were informed about the study and were aware of the ICU hospital pharmacist's presence on the ward. The method of data collection and medication order review by ICU hospital pharmacists was the same as during the baseline period. The detected prescribing issues and the recommendations, however, were discussed with the attending ICU physicians during the daily multidisciplinary patient review meeting instead of only with A-CdP. If consensus was reached between the ICU hospital pharmacist and the attending ICU physicians on a recommendation regarding a prescribing issue, then that issue was scored as a prescribing error and the medication order was corrected by the responsible attending ICU physician. If consensus could not be reached, the prescribing issue was not scored as a prescribing error and the medication order was regarded as appropriate. Our intention was to carry out the proposed activities every weekday.

### Outcome measures and definitions

The primary outcome parameter was the incidence of prescribing errors per 1,000 monitored patient-days. A prescribing error was defined as any prescribing issue, detected by the ICU hospital pharmacist during the medication review and agreed upon by the attending ICU physicians during the multidisciplinary patient review meeting, that may have caused or led to inappropriate medication use or patient harm while the medication was in the control of the healthcare professional or the patient [30].

The rate of consensus was defined as the percentage of recommendations agreed upon by the ICU physicians (intervention period) or A-CdP (baseline period) and the ICU hospital pharmacist. A monitored patient-day was defined as each patient day in the ICU during which the patient's prescribed medication was reviewed by the ICU hospital pharmacist.

The secondary outcome parameter was the number of prescribing errors that resulted in patient harm, preventable ADEs, NCC-MERP severity categories E, F, G, H and I, per 1,000 monitored patient-days. Patient harm was defined as temporary or permanent impairment of the physical, emotional, or psychological function or structure of the body and/or pain requiring intervention resulting from this impairment [30].

### Description of process costs and potential savings

Any deployment of resources and the related costs of the medication order review in the ICU by the ICU hospital pharmacists were included in the cost description. The duration of the ICU hospital pharmacist's medication order review and the duration of the subsequent discussions with ICU physicians during the multidisciplinary patient review meeting were recorded. The time spent by the ICU physicians during the discussions was also measured. The time investments related to training prior to the baseline period were discarded and were not included in the description of process costs. These costs were nonrecurring and negligible if divided over all monitored patient-days. The costs (€) were expressed per 1,000 monitored patient-days and adjusted for monetary inflation to the reference year 2006. The cost calculation of the medication review followed national costing guidelines for healthcare research [31]. In particular, unit costs for staffing and deployment of the ICU hospital pharmacists and ICU physicians were based on standardized salary costs (one salary level above the middle of the appropriate salary scale), additional costs for aggravating circumstances, and overhead costs.

In an attempt to quantify the economic benefits of prevented ADEs in the ICU, an estimation of potential savings was made using the costs of a preventable ADE derived indirectly from a study by Bates and colleagues [32]. A cumulative price index and the Organization for Economic Cooperation and Development-purchasing power parity of €0.867 for each US dollar (accessed March 2007) were used to make the calculations.

### Statistical analysis

Descriptive statistics were calculated for the analysis, including means, standard deviations, medians, and 25th and 75th quartiles. Subjects from the baseline population were compared with those from the intervention population using the unpaired Student *t *test or the Mann-Whitney U test for continuous data and using the chi-square test for categorical data. Two-sided Fisher's exact tests were used for the comparison of incidences of prescribing errors between the study periods. A multivariate, backward logistic regression analysis was applied to calculate odds ratios of finding a prescribing error by an ICU hospital pharmacist at least once during a patient's stay on the ICU for the selected patient characteristics. *P *< 0.05 was considered statistically significant. Computer software SPSS version 12.1 (SPSS Inc., Chicago, IL, USA) was used for the computations.

## Results

### Study population

Demographic characteristics of patients admitted during the baseline period (3 to 22 October 2005), during the first half of the intervention period (24 October 2005 to 25 February 2006) and during the second half of the intervention period (27th February to 30 June 2006) are shown in Table 2. The subset of patients reviewed during the second half of the intervention period had a significantly longer ICU stay than the subset of patients reviewed during the first half of the intervention period. No other significant differences were found between patient groups reviewed in the different periods of the study.

**Table 2:** Demographic characteristics of study patients

Characteristic	Baseline (*n *= 115)	Intervention (*n *= 1,058)		Statistics and *P *value
			
		First half (*n *= 573)	Second half (*n *= 485)	
Age (years)	63.22 ± 17.62	61.29 ± 15.49		*t *test, *P *= 0.212
		61.66 ± 15.26	60.86 ± 15.75	*t *test, *P *= 0.403
Male	42 (36.5)	376 (35.5)		Chi-square test, *P *= 0.834
		203 (35.4)	173 (35.7%)	Chi-square test, *P *= 0.935
APACHE II score	17.44 ± 6.80	18.12 ± 7.40		*t *test, *P *= 0.357
		18.30 ± 7.54	17.91 ± 7.24	*t *test, *P *= 0.392
Length of ICU stay (days)	2.06 (1, 5)	2.65 (1, 6)		Mann-Whitney U test, *P *= 0.920
		2.02 (0.9, 5)	2.85 (2, 6)	Mann-Whitney U test, *P *= 0.000
Acute admission	66 (57.9)	572 (54.1)		Chi-square test, *P *= 0.441
		309 (53.9)	263 (54.3)	Chi-square test, *P *= 0.893
Number of monitored days per admission	3.0 (2, 5)	3.0 (2, 6)		Mann-Whitney U test, *P *= 0.559
		3.0 (2, 6)	3.0 (2, 6)	Mann-Whitney U test, *P *= 0.824

Data presented as mean ± standard deviation, *n *or median (25th, 75th quartiles). APACHE, Acute Physiology and Chronic Health Evaluation; ICU, intensive care unit.

The ICU hospital pharmacists reviewed medication orders for the ICU patients during a total of 125 days (15, 67, and 43 days during the baseline period and the first and the second halves of the intervention period, respectively). In daily practice, an average of 3 days a week (range 1 to 5 days a week) was attainable for the ICU hospital pharmacists to carry out the described activities, resulting in 504 monitored patient-days during the baseline period and 5,901 during the intervention period (3,200 during the first half and 2,701 during the second half). The average time invested by the ICU hospital pharmacists was 3.1 hours a day during the baseline period (range 2 to 4 hours a day) and 2.5 hours a day during the intervention period (range 0.5 to 4.5 hours a day).

### Rate of recommendations and consensus

**Figure 2:**
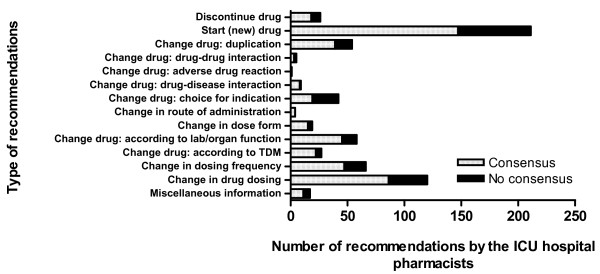
**Type and number of recommendations by intensive care unit hospital pharmacists during whole study period**. Recommendations were given during intensive care unit (ICU) patient review meeting. The results are divided into accepted (consensus) and not accepted (no consensus) recommendations. TDM, therapeutic drug monitoring.

During the entire study period, the ICU hospital pharmacists made 659 recommendations, of which consensus between the ICU hospital pharmacists and the attending ICU physicians was reached for 465 (71%). The rate of recommendations gradually decreased over the entire intervention period with the exception of a slight increase at the beginning of a training period of new residents in April 2006. The percentage of recommendations with consensus during the baseline period increased from 60 to 74% during the intervention period. For almost all types of recommendations, the rate of consensus was 60% or higher (Figure 2). Only for the recommendations related to choice of drug for an indication was the rate of consensus lower (45%). Examples of recommendations are presented in Table 3.

**Table 3:** Examples of ICU hospital pharmacist's recommendations and clinical consequences of prescribing errors scored during study.

Recommendation	Description and clinical consequence
Change drug order according to laboratory test/organ function	Ganciclovir intravenous dosage 5 mg/kg/48 hours too high. Recommended dosage according to renal function was 1.3 mg/kg/48 hours
	Consequence: renal failure and thus temporary harm to the patient that required prolonged hospitalization (Category F)
Change route of administration	Azathioprine in oral form was causing abdominal pain. This adverse reaction was not recognized in a timely manner. After switching to intravenous form the abdominal pain disappeared. Consequence: temporary harm to the patient that required intervention (Category E, moderate harm to a patient)
Change dosage	Phenytoin intravenous treatment was initiated with only a maintenance dose and without a loading dose.
	Consequence: an intervention was required to preclude harm to a patient (Category D).
Change drug because of drug-disease interaction	Patient with known liver function insufficiency was started on voriconazole (antifungal medication that is mostly metabolized by the liver).
	Consequence: an intervention was required to preclude harm to a patient (Category D)
Start drug	Unintended discontinuation of low-dose aspirin (patient's home medication) for 1 day
	Consequence: no harm to a patient (Category C)
Change dosage	Esketamine (anesthetic) 35 mg/hour (should have been 35 μg/hour) was ordered. This medication order was intercepted in the hospital pharmacy
	Consequence: no harm to a patient (Category B)
Start drug	The pharmacist proposed continuation of a statin during ICU admission. No consensus was reached with ICU physicians because of lack of evidence and the possible negative effects of the pleiotropic effect of statins. No error

ICU, intensive care unit.

### Effect of the intervention

The incidence of all prescribing errors, irrespective of their severity, was significantly lower during the intervention period compared with the baseline period: 62.5 versus 190.5 per 1,000 monitored patient-days, respectively - a difference of 127.9/1,000 (95% confidence interval (CI) = 89.3/1,000 to 166.6/1,000, *P *< 0.001). A further analysis of the intervention period, when subdivided into two halves, showed a significant decrease of all prescribing errors from 77.8 per 1,000 monitored patient-days during the first half of the intervention period to 44.4 per 1,000 monitored patient-days during the second half of the intervention period - a difference of 33.3/1,000 (95% CI = 20.9/1,000 to 45.9/1,000, *P *< 0.001) (Table 4).

**Table 4:** Reduction of incidence of prescribing errors per 1,000 monitored patient-days.

	Baseline	Intervention		Difference (95% CI)	*P *value	**Reduction (%)**^**a**^
					
		First half	Second half			
Prescribing errors	190.5	62.5		127.9 (89.3 to 166.6)	<0.001	67.1
		77.8	44.4	33.3 (20.9 to 45.9)	<0.001	42.8

CI, confidence interval. ^a^Two-sided Fisher exact test.

The incidence of prescribing errors that resulted in patient harm (preventable ADEs) per 1,000 monitored patient-days was 4.0 during the baseline period compared with 1.0 during the intervention period (*P *= 0.25). Only preventable ADEs in NCC-MERP severity categories E and F were found during the whole study. According to the Common Terminology Criteria for Adverse Events criteria, the two preventable ADEs found during the baseline period caused severe patient harm (abdominal spasms requiring morphine and increased liver function tests). Of the six preventable ADEs found during the intervention period, four caused severe patient harm (seizures, pancytopenia, hypoxia and hypotension) and two caused moderate patient harm (decreased creatinine clearance, abdominal pain). In comparison with the first part of the intervention period, the preventable ADEs decreased during the second half of the intervention period - with a rate difference of 1.9/1,000 monitored patient-days (95% CI = 0.4/1,000 to 3.4/1,000, *P *< 0.05).

The incidence of potentially harmful prescribing errors (potential preventable ADEs, NCC-MERP severity category D) per 1,000 monitored patient-days was 53.6 during the baseline period compared with 16.1 during the intervention period - a difference of 37.5/1,000 (95% CI = 17.0/1,000 to 57.9/1,000, *P *< 0.001). In comparison with the first half of the intervention period, the potentially harmful prescribing errors decreased from 19.7 to 11.8 per 1,000 monitored patient-days during the second half of the intervention period (*P *= 0.022).

**Figure 3:**
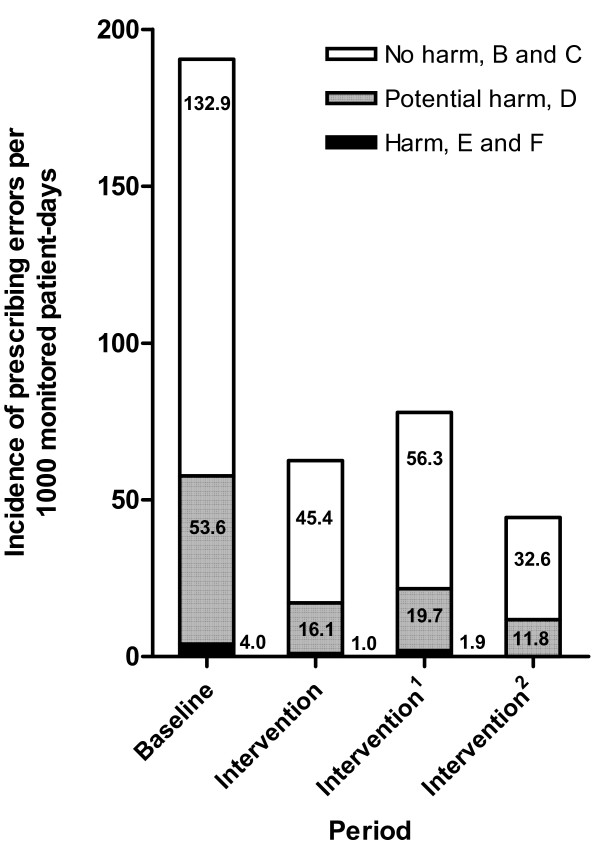
**Incidence of prescribing errors per 1,000 monitored patient-days, grouped by study period and severity**. The severity was scored according to The National Coordinating Council for Medication Error Reporting and Prevention Taxonomy of Medication Errors (categories B to F). Intervention^1 ^is the first half (first 4 months) of the intervention period; Intervention^2 ^is the second half (following 4 months) of the intervention period.

The incidence of prescribing errors that did not result in patient harm (NCC-MERP severity category B or C) per 1,000 monitored patient-days was 132.9 during the baseline period compared with 45.4 during the intervention period - a difference of 87.5/1,000 (95% CI = 55.2/1,000 to 119.8/1,000, *P *< 0.001). In comparison with the first half of the intervention period, the prescribing errors that did not result in patient harm decreased from 56.3 to 32.6 per 1,000 monitored patient-days during the second half of the intervention period (*P *< 0.001) (Figure 3).

**Figure 4:**
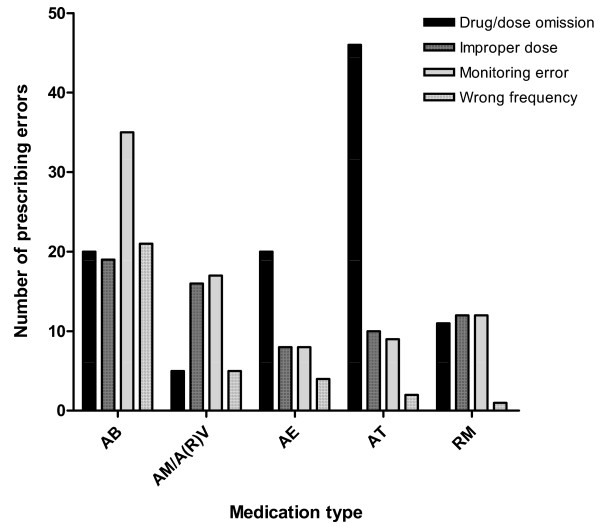
**Medication types with most prescribing errors found by intensive care unit hospital pharmacists**. Medication types with most prescribing errors found during the whole study period. The results are categorized by prescribing error type. AB, antibiotics; AM/A(R)V, antimycotics and anti(retro)viral medication; AE, antiepileptics; AT, antithrombotics; RM, respiratory medication.

The majority of prescribing errors were related to drug or dose omission errors, to monitoring errors (especially suboptimal therapeutic drug monitoring, suboptimal dosing according to renal and liver function and/or renal replacement therapy) and to improper dosage errors (31.6%, 25.4% and 18.5% of the total number of prescribing errors, respectively). Prescribing errors that resulted in patient harm (NCC-MERP severity category E or F) were found in the categories drug or dose omission error and monitoring error type (Figure 1). Figure 4 shows the types of drugs most frequently involved in the prescribing errors: antibacterials (23.4% of the total number of prescribing errors), drug therapies subjected to frequent changes, such as antithrombotics (14.8% of the total number of prescribing errors), and drugs less often prescribed in an ICU, such as antiepileptics (10.8% of the total number of prescribing errors).

The multivariate logistic regression analysis showed that acute admission and the APACHE II score were significantly associated with the chance of the ICU hospital pharmacist finding a prescribing error at least once during patient's ICU stay. The ICU hospital pharmacists found 2.1 more prescribing errors in acutely admitted patients than in electively admitted patients; and for every increase in the APACHE II score by 1 point, the ICU hospital pharmacists found 2.9% more prescribing errors (Table 5).

**Table 5:** Stepwise multiple logistic regression model of the likelihood of finding an error: final model

Variable	*P *value	Odds ratio (95% CI)
Age (years)	0.09	0.99 (0.98 to 1.00)
Weight (kg)	0.07	0.99 (0.98 to 1.00)
APACHE II score	0.01	1.03 (1.003 to 1.01)
Type of admission		
Elective		Reference category
Acute	0.00	2.12 (1.57 to 2.88)

APACHE II, Acute Physiology and Chronic Health Evaluation II; CI, confidence interval.

### Process costs and potential savings

Table 6 presents the costs of the medication review by the ICU hospital pharmacists and feedback to the IC-physician per 1,000 monitored patient-days during the intervention period. The total costs for the first half and for the second half of the intervention period amounted to €3,756 and €2,653, respectively. In the latter case, this is less than €3 per monitored patient-day. In Figure 3, we noted that the number of preventable ADEs per 1,000 monitored patient-days was reduced from 4.0 to 0 during this study. Using the data of Bates and colleagues [32], the total savings may thus have been between €26,312 and €39,808 per 1,000 monitored patient-days for medical and surgical patients, respectively. This would amount to savings of between €26 and €40 per monitored patient-day, depending on the mix of medical and surgical patients. Even if only one-half of these savings could be realized, one could at least expect a fourfold return on investment following the implementation of our ICU program for a hospital pharmacist.

**Table 6:** Total costs of medication

Intervention period	Clinical pharmacist			ICU physician			**Total costs**^**a**^
		
	**Unit costs**^**b**^	**Number of hours**^**a**^	**Costs**^**a**^	**Unit costs**^**b**^	**Number of hours**^**a**^	**Costs**^**a**^	
First half	€70	52.76	€3,693	€100	0.625	€62.5	€3,756
Second half	€70	37.21	€2,605	€100	0.481	€48.1	€2,653

A review by intensive care unit (ICU) hospital pharmacists and feedback to ICU physicians per 1,000 monitored patient-days. ^a^Per 1,000 monitored patient-days. ^b^Unit costs (gross salary costs per hour per specialist) are given per hour; for derivation of the unit costs, see Materials and methods.

## Discussion

Our study has shown that the designed on-ward participation program for a hospital pharmacist in the ICU increased medication safety on that ward.

Although a direct comparison with other studies is hampered by differences between clinical settings, study designs and outcome definitions, we found baseline error incidence rates and error reductions by an intervention in line with findings of other studies [4-12,14,20,22]. In our program, the participating ICU hospital pharmacists significantly reduced the number of all prescribing errors and those that resulted in patient harm (77% and 100% reduction, respectively). These results are comparable with the findings of Leape and colleagues [11] and Kaushal and colleagues [12], who showed a 66% reduction in preventable ordering ADEs per 1,000 patient-days and a 79.3% reduction in serious medication errors per 1,000 patient-days, respectively.

The difference between the intensity of our on-ward participation program and that of others is striking. In our program, the participating ICU hospital pharmacists spent on average 3 days per week and 2.5 hours per day in the ICU. The programs of Leape and colleagues and Kaushal and colleagues were much more extensive, mainly because of clinical pharmacist participation in ward rounds, physician staff meetings and nursing staff assistance, requiring half-time or even full-time commitment in case of pediatric ICU patient care team [11,12]. Our findings suggest that with a less extensive but highly focused on-ward medication order review program, a hospital pharmacist can also effectively reduce prescribing errors and related patient harm in an ICU. In spite of the limited time investment by ICU hospital pharmacists and in spite of the fact that our ICU physicians were not accustomed to on-ward hospital pharmacist's consultations, the high number of recommendations accepted by these physicians shows that ICU hospital pharmacist's recommendations were clinically relevant. Our results hold promise for hospital pharmacy settings where a full-time, on-ward commitment of a hospital pharmacist is not feasible, but on-ward participation is desirable from a medication safety perspective. Of course, more studies are required to confirm our findings.

The most important risks that emerge from our study can be categorized into patient characteristics, medication and prescribing processes. Acutely admitted patients and patients with high APACHE II scores appeared to be most at risk for prescribing errors. Although the length of ICU stay was significantly different between the two subsets of the intervention period, this characteristic is unsuitable as a predictor because the relationship between length of ICU stay and the likelihood of finding a prescribing error works both ways: the longer the ICU stay, the more risk there is a prescribing error will be made - but the reverse can also be true. The length of ICU stay was therefore not included in our model.

Medication risks were mostly associated with orders for antibiotics, for drugs less frequently prescribed by ICU physicians, such as antiepileptics, and for medication subjected to frequent changes, such as antithrombotics. The most prominent prescribing process-related risks were drug/dose omissions, improper dosing and lack of monitoring. Overall, these risks largely match the risk factors listed by Moyen and colleagues in their systematic review of medication errors in critical care [4]. We did not find all risks identified by these authors, however, probably due to differences in settings, study designs and outcome definitions. The prescribing-related and patient-related risks determined in the present study suggest that our participation program could be made more efficient in the future if the ICU hospital pharmacist reviewed medication orders with a focus on the most frequently occurring errors. Of notable interest is also a slight increase in recommendations at the start of a training period for new residents, suggesting that an additional ICU hospital pharmacist effort at that moment might be desirable.

Potentially, the additional costs of the participation program described in this study are well outweighed by the savings resulting from more appropriate drug therapy. Once the monitoring of prescribing by an ICU hospital pharmacist is well established, a ninefold to 13-fold return on investment seems feasible, depending on the mix of medical and surgical patients. Even if only one-half of this figure could be realized, the resulting fourfold to sixfold return on investment would still be attractive from a societal perspective. Moreover, these cost savings are likely to be underestimated as they only result from the reduction of preventable ADEs (prescribing errors in NCC-MERP severity categories E and F). There is no generally accepted way of calculating the cost savings arising from the reduction of potentially preventable ADEs (NCC-MERP severity category D) and the prescribing errors in NCC-MERP severity categories C and B that were intercepted on time by the ICU hospital pharmacist (see Table 4). These results thus substantiate the acceptability of our on-ward participation program.

The current reimbursement structure by the Dutch government for ICU hospitalizations, however, is based on a fixed price per day structure. It is clear that such a structure acts prohibitively on quality improvements, like the on-ward deployment of hospital pharmacists, regardless of whether it is likely to improve drug therapy goals set by the ICU physicians. In addition, the potential savings from a societal perspective are not at all represented by this 'fixed price per day' reimbursement structure.

### Limitations

Our study has several limitations. First, it was performed in only one ICU, which could reduce generalization of our findings to other clinical settings. However, because the reduction of prescribing errors and related harm was substantial in our study, and those results were in line with earlier published findings, it is highly probable that comparable beneficial effects will be achieved when similar on-ward participation programs will be implemented in other hospitals with similar ICU and hospital pharmacy settings.

Second, our study was not designed as a randomized controlled trial, and therefore could be biased by a large number of causes. However, such a refined study design is very time consuming, and is mostly chosen for interventions of which the effects have already been explored by studies with less sophisticated designs. To our knowledge, this is the first study that has investigated the effect of an on-ward participation program designed for a hospital pharmacist in a Dutch ICU; our priority was therefore to conduct a practical study to explore the potential added value of this approach to medication safety on this ward.

## Conclusions

Our on-ward participation program for a hospital pharmacist in a Dutch ICU resulted in clinically relevant recommendations by the ICU hospital pharmacist and in significant reduction in prescribing errors and preventable ADEs. The results of this study provide a sound justification for an on-ward involvement of hospital pharmacists in ICUs in clinical settings similar to ours, and can be used to convince policy-makers to invest in development and implementation of such programs on wards where patient care is very complex and medication use is error-prone.

## Key messages

• The present study is the first study in The Netherlands evaluating the effect of an on-ward program for an ICU hospital pharmacist on prescribing errors and related patient harm.

• The incidences of prescribing errors and related patient harm were reduced significantly.

• Even in settings with less resources and not well established on-ward clinical pharmacy services, a hospital pharmacist can play an important role in enhancing medication safety on the ICU wards.

• By evaluating the types of prescribing errors found and by analyzing selected patient characteristics, we were able to identify risks for prescribing errors. This risk stratification will help us to improve our ICU on-ward program in the future and could make the program more efficient and effective.

## Abbreviations

ADEs: adverse drug events; APACHE: Acute Physiology and Chronic Health Evaluation; CI: confidence interval; ICU: intensive care unit; NCC-MERP: National Coordinating Council for Medication Error Reporting and Prevention; PDMS: patient data management system.

## Competing interests

The authors declare that they have no competing interests.

## Authors' contributions

JEK was responsible for data collection, analysis of the data, statistical analysis and drafting of the manuscript. RK and HJvK were responsible for conceiving the study, data collection and critical revision of the manuscript. A-CdP and LL-A-H facilitated the data collection and were responsible for critical revision of the manuscript. MGD helped with statistical analysis, drafting of the article and critical revision of the manuscript. MBV was responsible for conceiving the study and critical revision of the manuscript. SMS was responsible for conceiving the study, helped to draft the manuscript and was responsible for critical revision of the manuscript. All authors read and approved the final manuscript.
